# Chaotic Boltzmann machines

**DOI:** 10.1038/srep01610

**Published:** 2013-04-05

**Authors:** Hideyuki Suzuki, Jun-ichi Imura, Yoshihiko Horio, Kazuyuki Aihara

**Affiliations:** 1 Institute of Industrial Science, The University of Tokyo, Tokyo 153–8505, Japan; 2 Graduate School of Information Science and Engineering, Tokyo Institute of Technology, Tokyo 152–8552, Japan; 3 Graduate School of Advanced Science and Technology, Tokyo Denki University, Tokyo 120–8551, Japan

## Abstract

The chaotic Boltzmann machine proposed in this paper is a chaotic pseudo-billiard system that works as a Boltzmann machine. Chaotic Boltzmann machines are shown numerically to have computing abilities comparable to conventional (stochastic) Boltzmann machines. Since no randomness is required, efficient hardware implementation is expected. Moreover, the ferromagnetic phase transition of the Ising model is shown to be characterised by the largest Lyapunov exponent of the proposed system. In general, a method to relate probabilistic models to nonlinear dynamics by derandomising Gibbs sampling is presented.

Boltzmann machines are a type of neural network model composed of stochastic elements. Since they were proposed more than twenty years ago, it has been demonstrated that they are capable of solving various problems such as optimisation problems^1^, repairing degraded images^2^, and learning interdependency among random variables^3^. For optimisation problems, convergence to the global optimum is guaranteed, provided that the system is annealed at a sufficiently slow rate^2^. For learning problems, parameter values for a Boltzmann machine that represent data distribution can be learned by a simple algorithm, which is theoretically sound and insightful^3^.

Although they are theoretically important, it is difficult to apply Boltzmann machines in their original form to real-world problems. The main difficulty is in their computation costs. For optimisation problems, the optimality is only assured theoretically for a extremely slow annealing schedule^2^, which is impractical. For learning problems, the learning algorithm requires lengthy computation to obtain equilibrium statistics^4^,^5^.

However, these difficulties do not necessarily mean that Boltzmann machines are not practically important. The capability of solving optimisation problems without prior knowledge of the problem structures is highly attractive. As for learning problems, many attempts have been made to increase computation speeds at the expense of the learning capability by restricting network structures and by using approximations in the learning algorithm. Even restricted versions of Boltzmann machines, such as restricted Boltzmann machines^6^ and deep Boltzmann machines^7^, combined with approximate learning algorithms, such as mean-field approximation^4^,^5^ and contrastive divergence^8^, achieve state-of-the art performance among various machine learning methods. This fact highlights the potential capabilities of Boltzmann machines.

Hardware implementation is one approach to enhance the computation speed of Boltzmann machines without degrading their capability. One of the difficulties in this approach is the generation of good random numbers, which is necessary to realise stochastic behaviour of the component units. Although thermal noise exists in electronic circuits, it is difficult to utilise this noise to precisely simulate the probabilistic behaviour of Boltzmann machines. Therefore, pseudo-random number generators^9^,^10^ or mechanisms to utilise physically controllable randomness, such as quantum effects^11^,^12^,^13^, are required in the circuit. Other previous studies on hardware implementation avoid randomness by using mean-field approximations.

In this paper, we propose a deterministic system that works as a Boltzmann machine, and show numerically that the system has computing abilities comparable to a Boltzmann machine. The apparently stochastic behaviour of the system is realised, without any use of random numbers, by chaotic dynamics that emerges from pseudo billiard dynamics^14^. Although the numerical simulation of the system is not efficient when calculated sequentially on ordinary digital computers, the proposed approach allows possibly efficient hardware implementation of a Boltzmann machine as a parallel distributed system. More generally, our approach presents a novel mechanism for biologically inspired information processing and analogue computing^15^,^16^,^17^,^18^,^19^,^20^.

## Results

### Chaotic boltzmann machines

A Boltzmann machine is a stochastic system composed of binary units interacting with each other. Let *s_i_* ∈ {0, 1} be the state of the *i*th unit in a Boltzmann machine composed of *N* units. Interactions between the units are represented by a symmetric matrix (*w_ij_*) whose diagonal elements are all zero. The states of the units are updated randomly as follows. First, a unit is selected randomly; let *i* be the index of the selected unit. The input *z_i_* to the *i*th unit is calculated as follows: 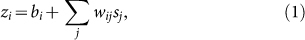
where *b_i_* represents a constant bias applied to the *i*th unit. The state of the *i*th unit is updated according to the probability 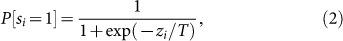
where *T* denotes the temperature of the system. By repeating this procedure for randomly selected units, the state ***s*** = (*s*_1_, …, *s_N_*) of a Boltzmann machine is updated sequentially. The obtained states eventually follow the Gibbs distribution 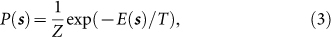
where the global energy *E* and the partition function *Z* are given by 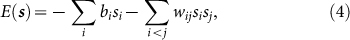

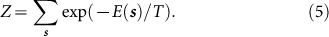


The procedure of state updates can be understood as Gibbs sampling, or as a Markov-chain Monte Carlo method.

Here, we propose a deterministic system that can simulate Boltzmann machines without any use of random numbers. In the system, the *i*th unit is associated with a state variable *x_i_* ∈ [0, 1], which we call the internal state of the unit. The internal state *x_i_* evolves according to the following differential equation 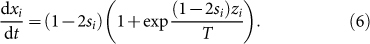
The states ***s*** of the units are updated by a deterministic rule, instead of the probabilistic rule described in equation (2). Specifically, the state *s_i_* ∈ {0, 1} of the *i*th unit changes when and only when *x_i_* reaches 0 or 1 as follows: 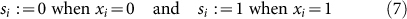
Note that regardless of the states of other units, the right-hand side of equation (6) is positive for *s_i_* = 0 and negative for *s_i_* = 1. Therefore, the internal state *x_i_* continues to oscillate between 0 and 1.

The differential equation (6) is designed from equation (2) so that it satisfies |d*x_i_*/d*t*| ∝ *P*[*s_i_*]^−1^. Let us assume that the states of other units are fixed. Then, according to equation (6), it takes (1 + exp(*z_i_*/*T*))^−1^ unit time for going up from *x_i_* = 0 to 1, and (1 + exp(−*z_i_*/*T*))^−1^ unit time for going down from *x_i_* = 1 to 0. Hence, *x_i_* oscillates between 0 and 1 with the period of 1 unit time, and so does *s_i_*. Therefore, the probability of observing *s_i_* = 1 is given by (1 + exp(−*z_i_*/*T*))^−1^, which is consistent with equation (2). However, since the states of other units in the system actually change, it is not theoretically assured that the probability of observing a certain state in the proposed system is exactly the same as the original Boltzmann machine. In this paper, we present numerical evidence that the proposed system actually works as a Boltzmann machine.

As indicated by equations (6) and (7), the internal state ***x*** = (*x*_1_, …, *x_N_*) goes straight in the hypercube [0, 1]*^N^*, and its direction changes only at the boundary. Therefore, the dynamics can be regarded as a pseudo billiard^14^ in the hypercube. The billiard dynamics induces a Poincaré map on the boundary of the hypercube. Since it exhibits chaotic behaviour as shown below, we call this system a chaotic Boltzmann machine. Note that the system dynamics can be computed by simple arithmetic calculations, because the right-hand side of equation (6) is piecewise constant.

### Application to combinatorial optimisation problems

As an example for solving combinatorial optimisation problems, we applied the chaotic Boltzmann machine to maximum cut problems. Given an undirected network of *N* nodes whose edge weights are represented by a symmetric weight matrix (*d_ij_*), the maximum cut problem is to find a subset *S* ⊂ {1, …, *N*} that maximises Σ*_i_*_∈*S,j*∉*S*_*d_ij_*. To solve this problem, the parameter values of the Boltzmann machines are set as *b_i_* = Σ*_j_d_ij_* and *w_ij_* = −2*d_ij_*. When the energy is minimised, the solution of the maximum cut problem is given by the set of nodes taking on the state *s_i_* = 1. We used the problem sets provided along with the Biq Mac solver^21^, for which exact solutions are also provided.

For comparison, we used conventional (stochastic) Boltzmann machines having the same parameter values. We regard *N* iterations of Gibbs sampling as one unit time. Note that this does not mean that one unit time of a stochastic Boltzmann machine corresponds to that of a chaotic one. Since the mechanisms are different, the comparison of time is not straightforward.

**Figure 1:**
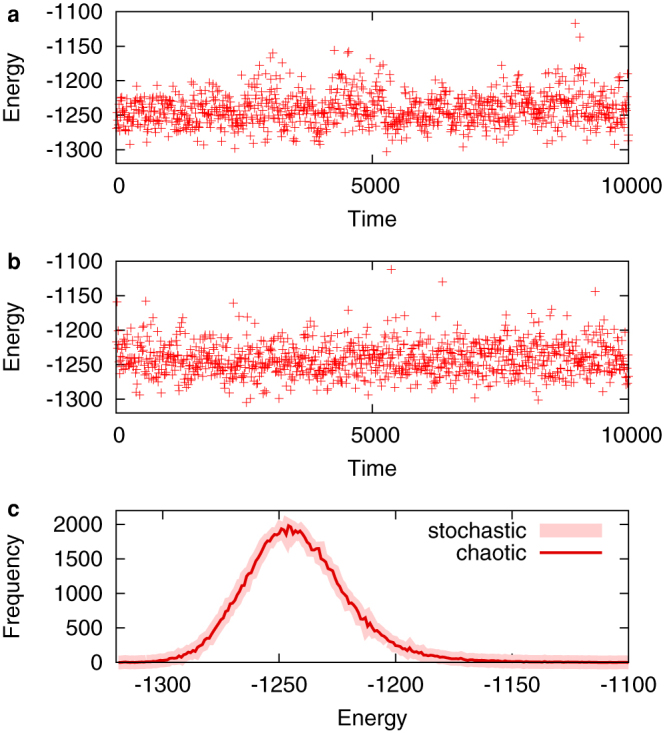
Behaviour of Boltzmann machines for a maximum cut problem (g05_100.0). Typical time evolutions of (a) chaotic and (b) stochastic Boltzmann machines for *T* = 128. Energy values are sampled every 10 unit time. (c) Histograms of the energy values sampled 100,000 times from chaotic (thin red line) and stochastic (thick pink line) Boltzmann machines.

**Figure 2:**
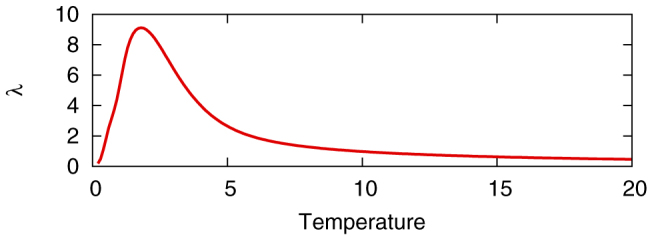
The largest Lyapunov exponent *λ* of the chaotic Boltzmann machine for a maximum cut problem (g05_100.0). For each temperature value, the Lyapunov exponent is calculated over 10,000 unit time for 50 different initial values. A peak is observed around *T* = 1.8.

The typical behaviour of chaotic and stochastic Boltzmann machines for a maximum cut problem is shown in Fig. 1. The temperature is fixed at a large value, *T* = max*_i_*Σ*_j_*|*w_ij_*|. The sampled energy values appear to fluctuate similarly in both types of models (Figs. 1(a) and (b)). The histograms of the sampled energy values coincide with each other (Fig. 1(c)). Figure 2 shows the largest Lyapunov exponent per unit time calculated for the Poincaré map induced on the boundary of the hypercube [0, 1]*^N^*. The Lyapunov exponent always takes positive values, thereby indicating chaos.

**Figure 3:**
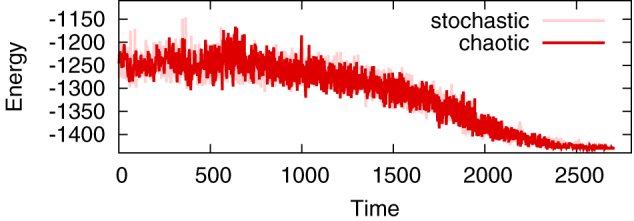
Simulated annealing of chaotic (red) and stochastic (pink) Boltzmann machines solving a maximum cut problem (g05_100.0). The energy values of the two models follow almost the same time course.

In order to solve the problem using Boltzmann machines, simulated annealing is applied, as shown in Fig. 3. The initial temperature is set to the same value as in Fig. 1, and it is multiplied by 0.95 every *N*/4 unit time. Annealing is terminated when the same energy value is sampled ten times consecutively. Table 1 shows statistics of the solutions obtained by chaotic and stochastic Boltzmann machines. For each of the datasets, simulated annealing is performed 100 times. For all the problems with *N* ≤ 100, optimal solutions are obtained. Overall, fairly good solutions (2–3% degraded from optima) are obtained without excessive tuning of the annealing schedule. The point to be noted here is that there is no significant difference between the chaotic and stochastic Boltzmann machines. A detailed comparison of the results has no meaning, because time cannot be directly compared.

It should be noted that there have been many studies that utilise chaotic dynamics for solving combinatorial optimisation problems^22^,^23^,^24^,^25^,^26^. We have presented novel chaotic dynamics that can be related to stochastic approaches to combinatorial optimisation problems.

### Application to the ising model

For application of Boltzmann machines to learning problems, a simple learning rule^3^ has been derived as gradient decent on the Kullback-Leibler divergence between the data distribution and the equilibrium distribution as follows: 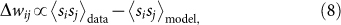
where 〈*s_i_s_j_*〉_data_ and 〈*s_i_s_j_*〉_model_ denote the expected values of *s_i_s_j_* at equilibrium state of the system in which the units are clamped to data vectors and unclampled, respectively. Therefore, for the learning process to work, it is essential to obtain faithful samples from equilibrium distributions.

To evaluate the applicability of the chaotic Boltzmann machines to such problems, we start from a simple example, the Ising model on a two-dimensional lattice, which has been extensively investigated. The Ising model is a simple model of ferromagnetism, and it can be regarded as a Boltzmann machine whose connections are limited to only neighbouring nodes in the lattices. Despite the simplicity of the model, it exhibits rich behaviour including phase transition with critical behaviour.

The Hamiltonian of the Ising model is given by 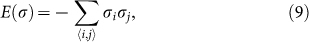
where summation is taken for every adjacent pair of the two-dimensional lattice. Note that here we use *σ_i_* ∈ {+1, −1}, instead of {0, 1}, following the convention in statistical physics. The differential equation of the chaotic Ising model used is given as follows: 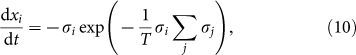
which is designed essentially in the same way as equation (6).

**Figure 4:**
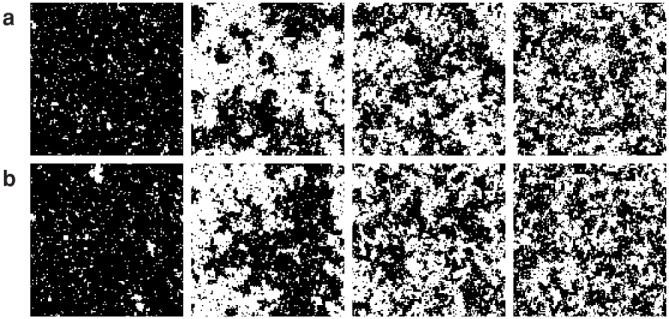
Snapshots of (a) chaotic and (b) stochastic Ising models on a two-dimensional lattice of size 128 × 128 with a periodic boundary condition for *T* = 2.1, 2.3, 2.5, and 2.7 (from left to right). The black and white dots represent the states +1 and −1, respectively.

**Figure 5:**
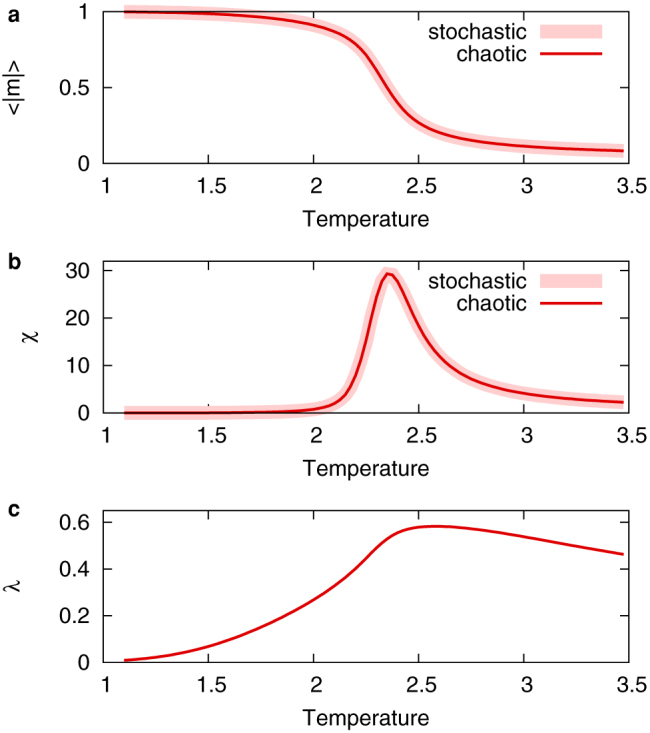
Statistics of chaotic (thin red lines) and stochastic (thick pink lines) Ising models on a two-dimensional lattice of size 24 × 24 with a periodic boundary condition. (a) The average absolute magnetisation per site 〈|*m*|〉 and (b) the magnetic susceptibility per site *χ*. The statistics are calculated during 100,000 unit time for 100 different initial values. (c) The largest Lyapunov exponent *λ* of the chaotic Ising model. Both the magnetic susceptibility and the largest Lyapunov exponent exhibit peaks corresponding to the ferromagnetic phase transition.

Figure 4 shows snapshots of the chaotic and stochastic Ising models. There appears to be no difference between the two models. As observed from Fig. 5, there is no difference in the following thermodynamic statistics: the average absolute magnetisation 〈|*m*|〉 = 〈|Σ*_i_σ_i_*|〉/*N* and the magnetic susceptibility *χ* = *N*(〈*m*^2^〉 − 〈|*m*|〉^2^).

The largest Lyapunov exponent is always positive in this case also. It should be noted that the largest Lyapunov exponents exhibit peaks in both Figs. 2 and 5(c). These peaks are analogous to those observed in neuron models coupled by gap junctions^27^,^28^. In Fig. 5, the peak corresponds to the ferromagnetic phase transition. This result is consistent with a previous study that also relates the peak to the synchronisation phase transition in the Kuramoto model^29^.

It should be noted that some deterministic discrete-time models that can simulate the Ising model have been proposed on the basis of the ideas of microcanonical ensembles^30^,^31^,^32^ and coupled map lattices^33^,^34^,^35^,^36^,^37^. The chaotic Ising model proposed in this paper has continuous-time billiard dynamics that is totally different from these two series of models.

## Discussion

It is intuitively understandable from equation (6) that a unit in a chaotic Boltzmann machine takes the states *s_i_* = 0 and 1 with the same probability as Gibbs sampling (equation (2)), because *x_i_* moves at a speed inversely proportional to the conditional probability as |d*x_i_*/d*t*| ∝ *P*[*s_i_*|***s***\*s_i_*]^–1^. However, considering all the interactions in the system, it is not trivial at all that the billiard ball moves following the joint probability of the Gibbs distribution (equation (3)). Although the results presented in this paper provide numerical evidence, further theoretical investigation of the dynamics from the viewpoints of both nonlinear dynamics and statistical mechanics is necessary. It is also important to characterise the differences in dynamical aspects of stochastic and chaotic Boltzmann machines. Chaotic Boltzmann machines can be a good example of a system whose macroscopic behaviour appears to be that of an equilibrium system, even though the microscopic behaviour is far from equilibrium^36^.

We have described a method to derandomise the Gibbs sampling of Boltzmann machines by using billiard dynamics. This approach seems applicable to a wider class of probabilistic models such as Markov random fields and graphical models. It can be extended to multi-valued random variables in the following two possible ways. One way is to extend the phase space of *x_i_* to [0, *S_i_*), where *S_i_* denotes the number of values of the *i*th unit. The internal state *x_i_* moves unidirectionally from 0 to *S_i_*, and the state *s_i_* is determined by 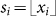. If the endpoints of the phase space are regarded as identical, *s_i_* takes values of 0, 1, …, *S_i_*−1 cyclically. Another way is to use a switched arrival system^38^ composed of *S_i_* tanks. In this case, *s_i_* switches probabilistically due to the chaotic behaviour of switched arrival systems; however, the system dynamics becomes non-invertible.

The numerical simulation of chaotic Boltzmann machines is slower than that of stochastic Boltzmann machines, because every time ***s*** is updated, the probabilities for all the units have to be calculated. As for stochastic Boltzmann machines, calculation is necessary only for the selected unit. Hence, it is impractical to use chaotic Boltzmann machines, instead of stochastic ones, on ordinary digital computers.

However, stochastic Boltzmann machines are not amenable to parallelisation. Even if all the nodes can communicate quickly with each other, state updates of stochastic Boltzmann machines must be carried out one by one, sequentially and exclusively; simultaneous updates of multiple units are not allowed except for special cases such as restricted Boltzmann machines and the Ising model, for which parallelisation depending on the specific network structures is possible. Due to the parallel distributed manner of information processing in the nervous system, neural network models appear to be easily parallelisable; however, this is not the case for stochastic Boltzmann machines. On the other hand, chaotic Boltzmann machines are defined as a dynamical system in which units evolve in parallel. Moreover, no random numbers are required. Therefore, efficient hardware implementation as a parallel distributed system is highly expected. Although there may be unexpected difficulties in hardware implementation of chaotic Boltzmann machines, this paper presents at least a novel mechanism for implementing Boltzmann machines that inherit the parallelism of neural networks.

Compared with stochastic Boltzmann machines, the units in chaotic Boltzmann machines are more like real neurons. The behaviour of internal states can be regarded as oscillators that interact with each other through discretised signals. The units are analogous to neurons in the brain that also show oscillatory behaviour and interact through digital signals, namely, neuronal spikes. Actually, a chaotic Boltzmann machine is similar to a neural network model of simplified hysteresis neurons^39^.

The functional roles of chaos in the brain have been discussed extensively^40^,^41^,^42^,^43^,^44^,^45^,^46^. Chaotic neural networks^41^ have been proposed as a simple neural network model that exhibits chaotic behaviour, and the chaotic dynamics has been shown to be effective when used for associative memory networks^43^,^47^ and for solving optimisation problems^22^,^23^. We have presented here another mechanism that induces chaotic behaviour in neural networks.

We have also shown that chaos in hybrid dynamical systems can be utilised for computing. Herding systems^48^ are an example that utilises the complex behaviour of piecewise isometries for machine learning; note that time discretisation of chaotic Boltzmann machines yields piecewise isometries. In general, hybrid dynamical systems with rich nonlinear dynamics are expected to form the possible components of future computers, as discussed in ref. 49. Chaotic Boltzmann machines can be a suitable candidate if they can be implemented efficiently in microelectronic circuits. Moreover, because a similar billiard dynamics can be implemented as a model of city traffic by using a switched flow system^50^, it may be possible to devise a physical mechanism that can realise chaotic Boltzmann machines. From the viewpoint of thermodynamics and reversible computing^51^,^52^,^53^, it is intriguing that the billiard dynamics of the chaotic Boltzmann machines is invertible.

In conclusion, we have proposed a chaotic dynamical system that works as a Boltzmann machine. We have shown numerically that chaotic Boltzmann machines have computing abilities comparable to the conventional ones. The proposed approach allows possibly efficient hardware implementation of a Boltzmann machine as a parallel distributed system. Moreover, chaotic Boltzmann machines are not merely an implementation of Boltzmann machines, as they have implications in various research fields including nonlinear dynamics, statistical physics, thermodynamics, computing, machine learning, and neuroscience.

## Methods

See ref. 50 for the derivation of the Poincaré map (spin-flip map) defined on the boundary of the hypercube.

## Author Contributions

H.S. conceived of the model, and analysed it. All the authors designed the research, and wrote and reviewed the manuscript, especially from the viewpoints of hybrid dynamical systems (H.S.), hybrid control systems (J.I.), circuit implementation (Y.H.), and analogue computing (K.A. and Y.H.).

## Figures and Tables

**Table 1:** Solutions of maximum cut problems obtained by chaotic and stochastic Boltzmann machines. For each dataset, the network size and the value of maximum cut are shown. The first and second line for each dataset show the statistics of the results obtained by chaotic and stochastic Boltzmann machines, respectively

	Problem		Obtained results		
dataset	size	opt.	max.	avg.	s.d.
g05_100.0	100	1430	1430	1415.62	13.65
			1430	1414.97	13.69
pw09_100.0	100	13585	13585	13447.51	133.99
			13585	13440.37	134.19
ising3.0-300_5555	300	8493173	8454235	8304704.46	88561.68
			8430298	8215987.23	98257.26
t2g20_5555	400	24838942	24798931	24346513.46	266841.24
			24791603	24138133.96	300049.99
t3g7_5555	343	28302918	28302918	27910192.78	317104.08
			28302918	27550104.43	423472.58
